# Assessment of the Dyeing Properties of the Pigments Produced by *Talaromyces* spp.

**DOI:** 10.3390/jof3030038

**Published:** 2017-07-05

**Authors:** Lourdes Morales-Oyervides, Jorge Oliveira, Maria Sousa-Gallagher, Alejandro Méndez-Zavala, Julio Cesar Montañez

**Affiliations:** 1 School of Engineering, University College Cork, Cork, Ireland; lourdesmorales@uadec.edu.mx (L.M.-O.); j.oliveira@ucc.ie (J.O.); M.deSousaGallagher@ucc.ie (M.S.-G.); 2 Department of Chemical Engineering, Universidad Autónoma de Coahuila. Saltillo, Coahuila 25280, Mexico; alejandro.mendez@uadec.edu.mx

**Keywords:** fungal pigments, dyeing properties, textiles, *Talaromyces*

## Abstract

The high production yields of pigments by *Talaromyces* spp. and their high thermal stability have implied that industrial application interests may emerge in the food and textile industries, as they both involve subjecting the colourants to high temperatures. The present study aimed to assess the potential application of the pigments produced by *Talaromyces* spp. in the textile area by studying their dyeing properties. Dyeing studies were performed on wool. The dyeing process consisted of three stages: scouring, mordanting, and dyeing. Two different mordants (alum, A; ferric chloride, F) were tested at different concentrations on fabric weight (A: 5, 10, 15%; F: 10, 20, 30%). The mordanting process had a significant effect on the final colour of the dyed fabrics obtained. The values of dyeing rate constant (*k*), half-time of dyeing (*t*_1/2_), and sorption kinetics behaviour were evaluated and discussed. The obtained results showed that pigments produced by *Talaromyces* spp. could serve as a source for the natural dyeing of wool textiles.

## 1. Introduction

Market trends to use natural colourants as food additives [1], natural dyes [2], functional foods [3], and cosmetic products [4] represent an opportunity for the application of natural pigments in several sectors of industry.

The possibility to exploit biological sources such as microorganisms for the production of natural pigments has been recommended [5]. However, the successful application of microbial pigments relies on high production yields, reasonable production costs and capital investment, regulatory approval, pigment characterisation, and stability to environmental factors such as temperature and light. Among the microorganisms with potential to produce a vast variety of pigments (*Monascus* homologues) is *Talaromyces* spp. (formerly *Penicillium* spp.) [6,7].

There has been a considerable effort in performing studies regarding the optimisation of the production of *Talaromyces* pigments [8,9,10,11]; however, there are only a few studies on the application of pigments produced by this strain [12,13].

*Talaromyces* pigments are thermally stable [14], implying that industrial application interests can emerge in the food and textile industries, as both processes involve subjecting the colourants to high temperatures.

Colourants used by the textile industry are predominantly synthetic; however, synthetic dyes are not environment-friendly, and recently the textile industry has been challenged to ensure compliance with environmental issues [15]. Natural colourants are environmentally friendly and biodegrade more quickly than synthetic dyes [16].

Additionally, natural pigments present properties of great interest in the textile industry, such as antibacterial properties. Fabrics can act as carriers of bacteria responsible for undesirable odours. It has been shown that *Talaromyces* pigment extracts possess antimicrobial properties [17]. These properties, along with the absence of toxicity [18,19], make them a valuable alternative as natural colourants in the textile industry.

However, natural colourants still face a huge disadvantage against synthetic colourants. Dyeing textile with natural colourants usually involves issues of limited shade range and lower fastness properties of the dyed fabrics. These problems have been overcome applying a pretreatment to the textile with mordants in order to create affinity between the fibre and the dye. Selection of mordant and concentration is important in natural dyeing processes, as mordants can increase the depth of shade or drastically alter the final colour of the dyed fabric [2].

The present study aimed to assess the potential application of the pigments produced by *Talaromyces* spp. in the textile area by studying their dyeing capacity.

## 2. Materials and Methods

### 2.1. Microorganism and Inoculum Preparation

*Talaromyces* spp. was used for the production of red pigments (Department of Food Science and Technology, Autonomous University of Coahuila, Saltillo, Coahuila). The purified strain had been previously isolated and characterised as *Penicillium purpurogenum* GH2 [20,21]. *Penicillium purpurogenum* has, however, been transferred to *Talaromyces* spp. [22]. The strain was maintained on PDA (Potato dextrose agar) slants at 4 °C and sub-cultured periodically. Inoculum was prepared in Erlenmeyer flasks (125 mL in capacity) containing 25 mL of Potato Dextrose Broth medium (PDB medium, ATCC medium:336), which were sterilised and inoculated with a spore suspension (1 × 10^5^ spores/mL) of *Talaromyces* spp. previously incubated for 5 days. The flasks were then incubated at 30 °C for 84 h in an orbital shaker (Innova 94, New Brunswick Scientific, Edison, NJ, USA) at 200 rpm [9,23].

### 2.2. Culture Media

The PDA medium was prepared with a concentration of 39.0 g/L (Bioxon, Mexico). The medium PDB medium was prepared by finely boiling 0.3 kg of diced potatoes in 500 mL of water until thoroughly cooked; then the potatoes were filtered through cheesecloth and water was added to the filtrate to complete a volume of 1.0 L. Finally, 20.0 g of glucose was added before sterilisation. The Czapek-dox modified medium [24] consisted of (g/L): d-xylose 15.0, NaNO_3_ 3.0, MgSO_4_·7H_2_O 0.5, FeSO_4_·7H_2_O 0.1, K_2_HPO_4_ 1.0, KCl 1.0 and ethanol 20.0.

### 2.3. Cultivation Conditions for Pigment Production

The initial pH of the Czapek-dox modified medium was adjusted to 5.0 before sterilising by using 0.22 μm sterile membranes (Millipore, Billerica, MA, USA). A mycelial suspension of *Talaromyces* spp. was inoculated at 10% (*v/v*) in 125 mL Erlenmeyer flasks containing 25 mL of medium. The inoculated flasks were incubated at 30 ± 2 °C in an orbital shaker (Innova 94, New Brunswick Scientific, Edison, NJ, USA) at 200 rpm for 6 days.

### 2.4. Pigment Extraction

The pigment extraction was performed according to the methodology reported by Méndez-Zavala et al. (2011) [25]. The pigment extract was centrifuged at 8000 rpm and at 4 °C for 20 min (Sorvall, Primo R Biofuge Centrifugation Thermo, Waltham, MA, USA) and then filtered through a 0.45 µm cellulose membranes (Millipore, Billerica, MA, USA) for the subsequent analysis of pigments. In this study, only extracellular pigments were considered. The analysis of red pigment production was conducted by measuring the absorbance of the filtered extract at 500 nm using a spectrophotometer (Cary 50, UV-Visible Varian, Palo Alto, CA, USA). This wavelength was selected by scanning the maximum sensitivity for the presence of the pigment (that is, the pigment absorbs maximum light at this wavelength). Red pigment extracts were stored in the dark at 4 °C before being used for the subsequent test.

### 2.5. Wool Dyeing Process

Wool fabric was bought at a local textile company. The dyeing process consisted of three stages: scouring [26], mordanting, and dyeing [12].

#### 2.5.1. Fabric Scouring

The fabric was first scoured to remove any impurities so that they would not interfere with the dyeing process [26]. Wool was rinsed with 100 mL of hot distilled water (60 °C) per gram of wool; then, 18 mL of neutral soap per litre of water was added, along with 25% *w*/*v* of sodium carbonate, taking into consideration the wool weight. The mixture was heated to 60 °C and the wool was constantly moved from top to bottom. Then, the fabric was washed with water at ambient temperature (six times).

#### 2.5.2. Fabric Mordanting

The fabric mordanting process was carried out using the pre-mordanting technique [12]. To assess the most appropriate mordant for *Talaromyces* spp. pigments, two different mordants were tested at different concentrations on the weight of fabric (Table 1). Concentrations were selected from literature according to recommended concentrations for these two mordants [27]. Wool was heated in mordant solutions at 70 °C for 1 h. Subsequently, the fabric was squeezed to remove excess liquid and then air dried at room temperature overnight.

### 2.6. Dyeing

The pre-mordanted wool was dyed with 40 mL of pigment extract (40:1, pigment extract per gramme of fabric) in a conical flask at 80 °C for 90 min. pH was not controlled. The colour of fabrics after dyeing was determinate by CIELAB colour coordinates using ColorEye XTS colorimeter (GretagMacbeth, Grand Rapids, MI, USA).

Pigment uptake was determined by measuring the optical density of the dye solution samples at a wavelength of 500 nm [27]. Percentage of pigment uptake (*q*, %) was calculated using the following equation:

(1) $$q=\frac{OD_{o}-OD_{i}}{OD_{o}}*100$$

where *OD_o_* is the initial optical density (500 nm) of the dye bath, and *OD_i_* is the optical density after dyeing at different sampling times (*i* min).

The first-order rate equation of Lagergren, which is one of the most widely used equations for the sorption of solute from a liquid solution, was employed to describe the pigment sorption kinetics. Rearrangement of the Lagergren model [28] was used for the variation of the adsorbed pigment as a function of time:

(2) qtqr=1−exp[(−ktr)(ttr)]1−exp(−ktr)

where *q_t_* and *q_r_* are the amount of adsorbed pigment at time *t* (min) and at equilibrium (%), respectively, *k* is the first-order rate constant (min^−1^), and *t_r_* is the longest time of the sorption process (min).

### 2.7. Data Analysis

The model parameters were estimated by non-linear regression analysis [29]. Results were analysed statistically by factorial ANOVA to test statistical differences (*p* < 0.05), followed by Tukey’s test at 5% probability for comparisons. Statistical and regression analyses were made with STATISTICA 7.1. (StatSoft, Inc., Tulsa, OK, USA, 2005,).

## 3. Results and Discussion

### Dying Properties of Pigments

The effect of the mordant used on L*, a*, and b* parameters are given in Table 2; L* represents a lightness value (a higher lightness value represents a lower colour yield). a* and b* represent the tone of the colour; positive values of a* and b* represent redder and yellowish tones, respectively.

Total colour difference ∆E* between the dyed fabrics and the undyed wool was calculated as:

(3) $$∆E^{*}=\sqrt[{}]{{\left(L_{U}^{*}-L_{D}^{*}\right)}^{2}+{\left(a_{U}^{*}-a_{D}^{*}\right)}^{2}+{\left(b_{U}^{*}-b_{D}^{*}\right)}^{2}}$$

where sub-index *U* and *D* represent undyed and dyed wool, respectively, for L*, a*, and b* values.

Fabrics were qualitatively perceived as different; the dyeing process using mordant F presented a red colour while the dyeing process using mordant A showed a red tending to brown colour. Colorimetric studies indicated that the tested mordants significantly affected the colour exhibited by the fabric. The dyed wool using mordant F presented a higher total colour difference from the undyed wool than the dyed wool using mordant A (Table 2). Higher values of a* were obtained with mordant F in comparison with mordant A, indicating that more reddish tones can be obtained using mordant F. Meanwhile, b* values indicated that more yellowish tones can be obtained with mordant A. These results were more evident with the hue values obtained; hue values near to 0 indicated the degree of redness while values near to 90 represented the level of yellowness. Moreover, the wool dyed using mordant F presented a higher saturation of colour (Chroma) and a higher yield (L*) than that dyed with mordant A.

Dyed fabrics obtained here presented a stronger shade (red colour) than the optimum conditions reported for the dyeing of cotton using pigments produced by different fungal strains (*Monascus purpureus*, *Isaria farinose*, *Emericella nidulans*, *Fusarium verticilliodes* and *Penicillium purpurogenum*) [12].

Similarly, a* and b* values were higher than those reported for the dyeing of leather and silk with *Talaromyces* pigments [11,30]; however, those results were attained without the addition of mordants.

Mordant concentration also affected the colour exhibited by the dyed fabric. When mordant F was used, the colour yield obtained increased with concentration. However, there was not a statistical difference between F2 and F3. It is noted that a more reddish colour (a*) was obtained by increasing the mordant concentration while there was not a statistical difference achieving a yellow tone (b*) between F2 and F3 or between F1 and F2. In terms of colour saturation, hue, and total colour difference, there was not statistical difference between F2 and F3 indicating that if an F concentration between 20 and 30% *w/w* is used during the mordanting process, the same colour can be obtained.

Different results were obtained using mordant A; colour yield increased by increasing mordant concentration. A more reddish colour (a*) was obtained using the highest concentration while the yellowish tone (b*) was not affected by concentration. Furthermore, the concentration did not affect colour saturation (Chroma). A higher degree of redness (Hue) and higher total colour difference (∆E*) was attained using the highest concentration of mordant A.

These results are consistent with those reported by Arroyo-Figueroa et al. (2011) [26], who stated that a colour variation in terms of CIELAB scale is obtained as function of mordant concentration during the dyeing process using natural colourants.

In technical dyeing using synthetic dyes, a total colour difference of 1 is accepted as a tolerable colour difference between dyeings. However, with the introduction of natural dyes into the textile dyeing process, a wider total colour difference can be accepted (∆E* = 2) [31]. Results demonstrated that, independent of the mordant and concentration tested, an acceptable colour difference between dyeings was obtained (∆E* = 0.71–1.70).

**Figure 1:**
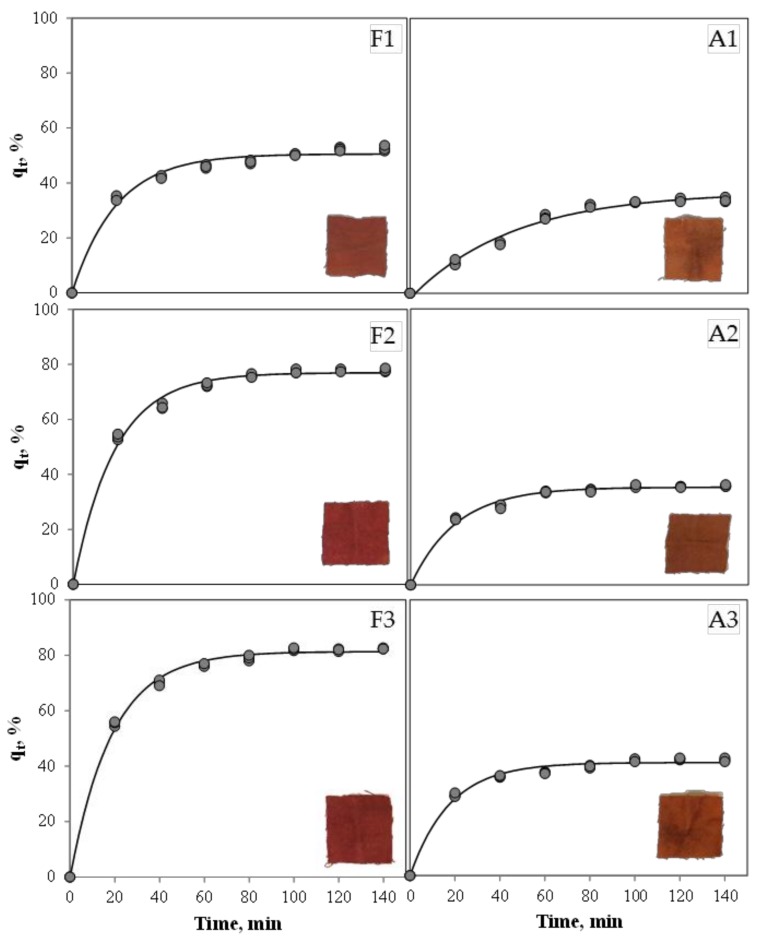
Effect of mordant type and concentration on pigment sorption on wool. Circles represent experimental data points and continuous lines represent the Lagergren model.

The effect of the dyeing process time on pigment uptake using different mordants at different concentrations is shown in Figure 1. It can be seen that dyeings with mordant F presented higher values of pigment uptake (42.54–82.44%) than mordant A (33.98–41.98%). In Table 3 are listed the kinetic parameters obtained with the regression analysis of Equation (2). When mordant F was used, pigment uptake at equilibrium (*q_r_*) increased as mordant concentration increased. Mordant A did not show a significant difference between concentrations A1 and A2, reaching the maximum pigment uptake with concentration A3. The higher pigment uptake here obtained (81.33 ± 0.43%) is similar to the maximum (80%) reported by Velmurugan et al. (2010) [12], which was achieved dyeing cotton with red pigments produced by *Monascus purpureus*.

Results showed that there was no statistical difference in terms of exponential rate (*k*) using mordant F at all concentrations and mordant A at concentrations A2 and A3. Only mordant A showed the lowest exponential rate at the lowest concentration studied.

In literature, sorption curves are classified into four zones (0 to 4) according to the *kt_r_* value; the kinetic behavior goes from no adsorption to drastic (Please see sorption kinetic behavior reported by Tseng et al. [28].

The time of half dyeing *t*_1/2_ is also used to express the time required for a fabric to adsorb half of the amount of the dye adsorbed at equilibrium (*q_t_*/*q_r_* = 0.5), calculated as:

(4) $$t_{1/2}=\frac{ln\left(2\right)}{k}$$

Table 4 lists the dimensionless rate constants (*kt_r_*), the half dyeing time values (*t*_1/2_), and the zone of kinetic sorption behavior presented for the mordants studied at three concentration levels.

Results showed that most of sorption kinetic curves fell into zone III (Kinetic curve type, rapidly rise; kinetic behavior, quick), while only the dyeing process using A1 fell into zone II (Kinetic curve type, continuously rising; kinetic behavior, good).

The *t*_1/2_ values obtained were in range with those reported for the optimised conditions for dying wool (*t*_1/2_ = 13.1 min) with natural anthraquinones dyes produced by the fungus *Fusarium oxysporum* [32]. The *t*_1/2_ values are relatively short in comparison with the longest time of the sorption process (*t_r_*), which could result in undesired colour yields. Thus, the above kinetics are useful to establish a target value (*q_t_/q_r_* > 0.5) to assist the engineering design and optimisation of the dyeing process of wool by *Talaromyces* pigments.

## 4. Conclusions

The above results showed the feasible potential applications of the pigments produced by *Talaromyces* spp.

These pigments could serve as a useful alternative source for the natural dyeing of wool textiles. High values of pigment uptake on the fabric were obtained. The values of dyeing rate constant (*k*), half-time of dyeing (*t*_1/2_) and sorption kinetics behaviour compare well with other natural dyes used for dyeing textile. However, the process still needs to be optimised in terms of dying process conditions (pigment concentration, pH, temperature).

It is concluded that the mordant used plays an important role in the dying process. Colorimetric studies showed that it is possible to attain a strong red shade; therefore, these pigments can compete with other synthetic or natural red dyes.

From this point onward, full characterisation of the molecules, toxicity studies, and more in-depth analyses such as molecular interactions between the dye and the fabric are worth tfurther investigation.

## Figures and Tables

**Table 1:** Mordants studied for the pre-mordanting process and selected concentrations. Codes were assigned.

Mordant	Concentration, % *w*/*w*	Code
Ferric chloride	10	F1
	20	F2
	30	F3
Alum	5	A1
	10	A2
	15	A3

**Table 2:** L*, a*, b* values of dyed wool.

Experiment	Colour Coordinates					
	L*	a*	b*	Chroma	Hue	∆E*
Wool	93.89 ± 1.16	0.44 ± 0.22	5.15 ± 1.47	5.17 ± 1.47	84.94 ± 2.63	0.00
F1	31.39 ± 1.11 ^b^	24.35 ± 0.44 ^c^	21.01 ± 0.20 ^c^	32.16 ± 0.23 ^b^	40.79 ± 0.76 ^b^	68.78 ± 0.96 ^b^
F2	28.20 ± 0.64 ^a^	27.62 ± 0.68 ^b^	20.61 ± 0.41 ^bc^	34.47 ± 0.64 ^a^	36.73 ± 0.78 ^a^	72.76 ± 1.70 ^a^
F3	27.53 ± 0.65 ^a^	28.91 ± 0.35 ^a^	21.87 ± 0.81 ^b^	36.25 ± 0.25 ^a^	37.10 ± 1.34 ^a^	74.14 ± 0.90 ^a^
A1	44.47 ± 0.68 ^e^	14.84 ± 0.15 ^e^	27.34 ± 0.32 ^a^	31.11 ± 0.34 ^d^	61.50 ± 0.21 ^d^	56.07 ± 0.71 ^d^
A2	42.48 ± 0.43 ^d^	15.07 ± 0.24 ^e^	27.83 ± 0.25 ^a^	31.65 ± 0.13 ^d^	61.56 ± 0.57 ^d^	58.09 ± 1.65 ^d^
A3	37.08 ± 0.56 ^c^	17.14 ± 0.35 ^d^	27.76 ± 0.14 ^a^	32.63 ± 0.18 ^d^	58.32 ± 0.57 ^c^	63.40 ± 1.52 ^c^

Different letters in each column indicate significant differences (Tukey’s post-hoc comparison, *p* < 0.05).

**Table 3:** Kinetic parameters and goodness of fit of the data.

Experiment	*q_r_*, %	*k*, min^−1^	*R*^2^
F1	50.56 ± 0.57 ^c^	0.0504 ± 0.0028 ^a^	0.98
F2	76.99 ± 0.55 ^b^	0.0542 ± 0.0021 ^a^	0.99
F3	81.33 ± 0.43 ^a^	0.0537 ± 0.0015 ^a^	0.99
A1	36.91 ± 0.82 ^e^	0.0218 ± 0.0011 ^b^	0.98
A2	35.40 ± 0.33 ^e^	0.0497 ± 0.0023 ^a^	0.99
A3	41.02 ± 0.39 ^d^	0.0569 ± 0.0030 ^a^	0.99

Different letters in each column indicate significant differences (Tukey’s post-hoc comparison, *p* < 0.05).

**Table 4:** Sorption kinetic parameter (*kt_r_*), *t*_1/2_ and zone of the sorption behavior.

Experiment	*kt_r_*	*t*_1/2_, min	Zone
F1	7.06	13.74	III
F2	7.58	12.79	III
F3	7.51	12.92	III
A1	3.04	31.84	II
A2	6.95	13.95	III
A3	7.97	12.17	III
